# Ruptured hemiarch and descending thoracic aorta aneurysm: hybrid treatment

**DOI:** 10.1186/1749-8090-7-66

**Published:** 2012-07-10

**Authors:** Alberto Settembrini, Daniela Mazzaccaro, Silvia Stegher, Maria Teresa Occhiuto, Giovanni Malacrida, Giovanni Nano

**Affiliations:** 1 First Unit of Vasular Surgery, Policlinico San Donato, Milano, Italy; 2 Postgraduate School in Vascular Surgery, Milano, Italy; 3 Università di Milano, Milano, Italy

## Abstract

Ruptured aortic arch aneurysm is a life threatening disease. Surgical repair has an high perioperative mortality rate and totally endovascular treatment is a challenge. Hybrid repair has been proposed as a valuable approach. We report the case of a patient with a contained rupture of aortic arch aneurysm. We treated him with a debranching of supraortic vessels with carotid-carotid and carotid-subclavian bypass and deployment of two enodgrafts in two different times. We consider hybrid treatment for arch and hemiarch a feasible option for aortic arch aneurysms in non emergent and in an emergency setting with an improvement in perioperative morbidity and mortality.

## Background

Ruptured aortic arch aneurysm has a high mortality rate [1]. Management of this lesions is a challenge. Surgical repair is still an invasive procedure requiring arch replacement with hypo- thermic circulatory arrest and emergency procedure is associated with high periprocedural mortality rate (7-17%) and neurological complications (4-12%) [2,3]. Endovascular stent-graft placement is safe and well standardized treatment in pathologies of descending aorta, also in emergency, but we have no many series about its use for aortic arch and hemiarch; if supra- aortic branches are involved, the placement of endovascular stent-graft requires fenestrations or surgical approaches to maintain cerebral perfusion [4,5].

We describe a case of a rupture of aortic hemiarch aneurysm treated with hybrid procedure.

## Case presentation

**Figure 1:**
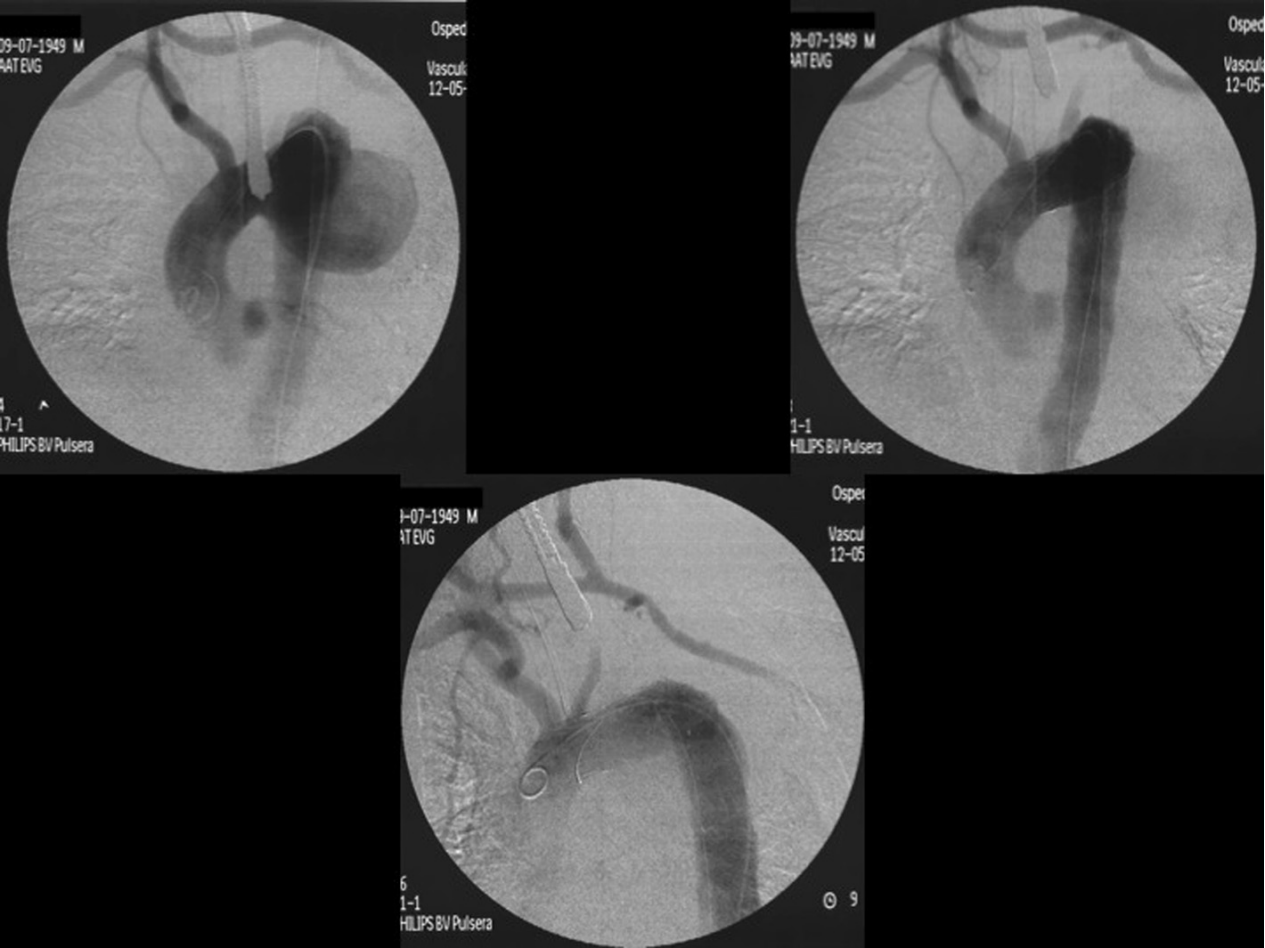
Deployment of the endograft and carotid-carotid-left subclavian bypass.

**Figure 2:**
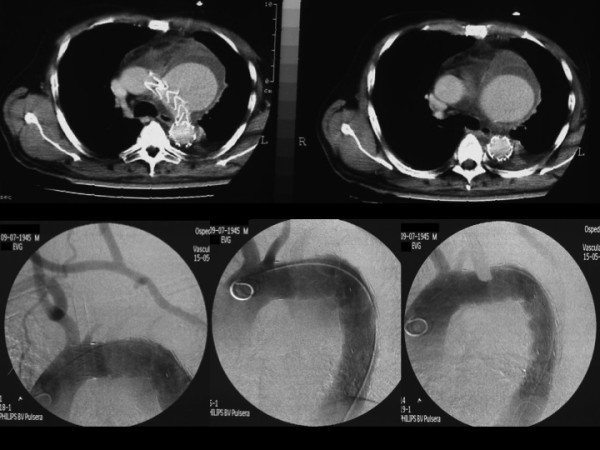
CT and angio images of displacement of the graft and enlargement of the sac.

A 60 years old man affected by hypertension, hypercolesterolemia and chronic obstructive pulmonary disease (COPD), went to a hospital with a constrictive chest pain. He was haemodynamically stable and he did not have any signs of myocardial infarction (troponine was negative as electrocardiogram). So a CT angiography was performed, revealing a rupture of an aneurysm of left aortic hemiarch measuring a diameter of 8.8 cm. Due to the pathology and for haemodynamic stability, the patient was sent to our institution. When he arrived he was troubled and suffering. At clinical examination we did not find any pathological signs at the thorax and the abdomen; femoral and peripheral pulses were valid. There were no neurological deficits. At laboratory exams he had moderate anemia (10.2 g/dl), normal creatininemia (0.90 mg/dl) and tachycardia (100 beats) with right bundle branch block at electrocardiogram. A new CT angiography showed the contained rupture of the aneurysm. We evaluated the aneurysm was in zone 2, according to Ishimaru classification [6], of the arch, but it would be changed into a zone 1 for a safer deployment of the graft. The study of CT angio showed a proximal neck diameter of 32 mm and a distal landing zone of 28 mm with a short proximal neck that required a surgical debranching. Under general anaesthesia and electroencephalogram monitoring, a debranching of supraortic vessels was performed, avoiding median sternotomy; extranatomic carotid-carotid right to left bypass, with a retropharyngeal course, and a left carotid to subclavian bypass were done, followed by endovascular repair of the aneurysm with deployment of endoprosthesis Relay-Bolton^TM^ 32/155 through femoral percutaneous approach. (Figure 1) There were no endoleaks at the completion angiography without any complication in puncture’s site. The patient was referred to Intensive Care Unit (ICU). In the first postoperative day the patient was extubated. He had no nerve injuries and neurological asymptomatic and vital signs were within normal limits. During the 2nd postoperative day he was clinically stable, but a left supraclavicular haematoma had grown up and chest pain was worsen with a diffused pulsatility in the left hemithorax. A CT angiography showed a new rupture of the aneurysm due to a type Ia endoleak for a dislocation of graft (Figure 2). The patient was taken to operating room to correct the lesion with deployment of endoprosthesis Relay^TM^ 36/190 with free flow on innominate artery. Again, no endoleaks at the end of the procedure.

**Figure 3:**
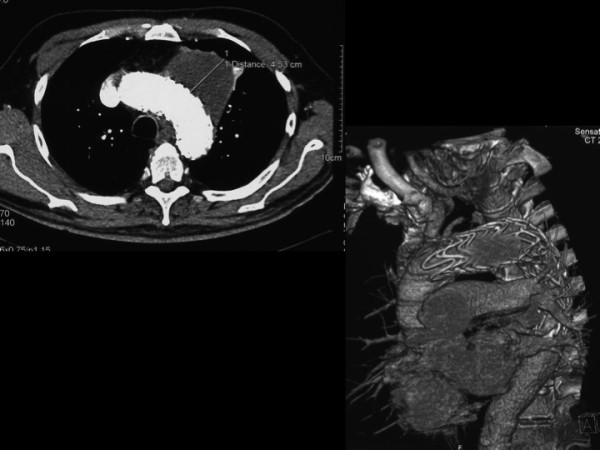
CT control after second operation: correct position of the graft and no leaks.

The patient was discharged in 17th postoperative day and CT angiography was performed: it showed a complete exclusion of the sac. The CT angiography follow up was strict at 2, 6 and 36 months showing a progressive shrinkage of the sac (now the diameter is 4.63) (Figure 3). Echocolordoppler evaluation revealed the normal patency of supraortic bypasses at 36 months.

## Discussion

The future of treatment of aortic arch pathologies is unclear. Since few years ago gold standard in the treatment of aortic arch pathology was surgery with cardio-pulmonary bypass and hypotermic cardiac arrest, but it carried a high mortality and morbidity with a significant incidence of neurologic injury. A totally endovascular solution for aortic arch is hard for involvement of supraortic trunks and the necessity of fenestration on the grafts or using chimney technique [7].

Recently, an hybrid treatment with a combined open and endovascular procedure has emerged and it has improved the chances in the treatment of pathologies of aortic arch, especially in the high-risk patient or in an emergent setting [8]. This technique consists of a surgical approach for revascularization of supra-aortic great vessels (using a supraortic transposition or debranching with bypasses) and endovascular deployment of an endograft.

This treatment allows both the preservation of the cerebral circulation and an optimal proximal landing zone and sealing of endovascular graft [8].

The rationale of the development of hybrid repairs is based on reduction of perioperative diseases avoiding cardio-pulmonary bypass and hypotermic cardiac arrest [7].

In literature we found different types of hybrid treatment of aortic arch pathology, but we did not find anything about the emergent treatment of a rupture hemiarch aneurysm. Szeto described a classification of hybrid repair dividing it into three types of repair based on aneurysm anatomy and landing zone suitability. But all the types require a multiple stage treatment and are long to do, so that they can’t be performed in emergency [7].

Our zone 2 ruptured lesion needed a supraortic reconstruction both to have a better landing zone and to avoid neurological adverse events. In emergency we did not have time to perform a multiple stage treatment because in this case we were between two problems: excluding the sac to avoid a worse evolution of the rupture and preventing any neurological accidents. We needed a fast surgical debranching for supraortic trunks to place safely the endograft [8].

Recently Joyeux et al. described a temporary extra anatomic bypass from femoral artery to supraortic trunks during the deployment of endograft and before performing sternotomy and debranching [9]. It could be another option for hybrid procedure but in our case surgical carotid-carotid bypass appears as a correct choice because we avoided the sternotomy, we allowed a patency of supraortic vessels without any risk of cerebral accident and we performed a well standardized and quite easy surgical intervention [4].

International guidelines suggest give some recommendation for the left subclavian artery (LSA) management and, in case of emergency, suggest that revascularization should be individualized and addressed. Our patient was haemodinamically stable with a tamponade rupture and we decided to perform the LSA revascularization considering how many affections could overcome if we did not perform it: neurological affections, the lack of preoperative evaluation on which vertebral artery was dominant and the steal syndrome [10]. We thought the risk of all of these affections could be increased by the emergency of intervention and the weak stability of the haemodynamic conditions [10,11].

During the deployment, in both procedures, pressure control was very precise keeping values below 110 mmHg and a lowering cardiac beats (the mean value has been 90 bpm). However, about endograft, it is important to remember they are not designed for the deployment in aortic arch. In our case we had a displacement of the graft after the first operation. The graft used for aortic arch aneurysm are designed for descending aorta and so they are tubular and tend to straighten to come back to their natural shape. Further improvement are needed in the future to optimize the use of these graft for aortic arch lesions [7,12].

## Conclusion

Emergent hybrid treatment for aortic hemiarch is not still standardized and there is not a widespread use of this kind of treatment. We report our first experience with hybrid, combining supra-aortic branch trans position with endovascular stent-grafting in emergency. Currently the hybrid treatment for arch and hemiarch is evolving with an improvement in perioperative morbidity and mortality in non emergent cases in high risk patients [1,13].

Even though this is just a case report and data are still limited and larger series are required to extend hybrid procedure to lower risk patients in non emergent cases, we deem that in case of rupture of aortic hemiarch aneurysm hybrid procedure could be a safe and effective treatment [14].

## Consent

Our Institution and the patient provided Clinical Consent for the publication of this case.

## Competing interest

All the authors declare that they have not competing interest.

## Author’s contribution

AS: concept and design, writing the article. DM: critical revision of the article. SS: analysis and interpretation. MTO: critical revision. GM: final approval of the article. GN: obtaining funding, final approval of the article. All authors read and approved the final manuscript.

## References

[B1] Jonker FH Trimarchi S Verhagen HJ Meta-analysis of open versus endovascular repair for ruptured descending thoracic aortic aneurysm J Vasc Surg 2010 51 1026 1032 10.1016/j.jvs.2009.10.103 20347700

[B2] Harrington DK Walzer AS Kaukuntla H Selective antegrade cerebral perfusion attenuates brain metabolic deficit in aortic arch surgery: a prospective randomized trial Circulation 2004 110 II231 II236 15364868 10.1161/01.CIR.0000138945.78346.9c

[B3] Westaby S Katsumata T Vaccari G Arch and descending aortic aneurysms: influence of perfusion technique on neu- rological outcome Eur J Cardiothorac Surg 1999 15 180 185 10.1016/S1010-7940(98)00310-8 10219551

[B4] Czerny M Fleck T Zimpfer D Combined repair of an aortic arch aneurysm by sequential transposition of the supra-aortic branches and endovascular stent-graft placamento J Thorac Cardiovasc Surg 2003 126 916 918 10.1016/S0022-5223(03)00222-8 14502197

[B5] Gottardi R Funovics M Eggers N Supra-aortic Transposition for Combined Vascular and Endovascular Repair of Aortic Arch Pathology Ann Thorac Surg 2008 86 1524 1529 10.1016/j.athoracsur.2008.06.075 19049743

[B6] Mitchell RS Ishimaru S Ehrlich MP First International Summit on Thoracic Aortic Endografting: roundtable on thoracic aortic dissection as an indication for endografting J Endovasc Ther 2002 9 98e105 12166849

[B7] Szeto W Bavaria J Hybrid Repair of Aortic Arch Aneurysms: Combined Open Arch Reconstruction and Endovascular Repair Semin Thorac Cardiovasc Surg 2009 21 347 354 10.1053/j.semtcvs.2009.11.007 20226349

[B8] Kang WC Shin EK Ahn TH Combined Open and Endovascular Repair for Aortic Arch Pathology Korean Circ J 2010 40 399 404 10.4070/kcj.2010.40.8.399 20830254 PMC2933465

[B9] Joyeux F Canaud L Hireche K Temporary extra-anatomic brain perfusion followed by total rerouting of the supra-aortic vessels for hybrid repair of a ruptured aortic arch aneurysm J Vasc Surg 2011 54 1145 1147 10.1016/j.jvs.2011.03.271 21658890

[B10] Melissano G Civilini E Bertoglio L Results of Endografting of the Aortic Arch in Different Landing Zones Eur J Vasc Endovasc Surg 2007 33 561e566 17207648 10.1016/j.ejvs.2006.11.019

[B11] Matsumura JS Lee WA Mitchell RS Farber MA Murad MH Lumsden AB Greenberg RK Safi HJ Fairman RM Society for Vascular Surgery, The Society for Vascular Surgery Practice Guidelines: management of the left subclavian artery with thoracic endovascular aortic repair J Vasc Surg 2009 50 5 1155 1158 10.1016/j.jvs.2009.08.090 19878791

[B12] Holt P Johnson C Hinchliffe R Outcomes of the endovascular management of aortic arch aneurysm: implication for management of left subclavian artery J Vasc Surg 2010 51 1329 1339 10.1016/j.jvs.2009.10.131 20303695

[B13] Ryu YG Choo SJ Lim JY Hybrid Procedure for a Traumatic Aortic Rupture Consisting of Endovascular Repair and Minimally Invasive Arch Vessel Transposition without Sternotomy J Korean Med Sci 2010 25 142 144 10.3346/jkms.2010.25.1.142 20052360 PMC2800019

[B14] Choi BK Lee HC Lee HW Succesful treatment of ruptured aortic arch aneurysm using a hybrid procedure Korean Circ J 2011 41 469 473 10.4070/kcj.2011.41.8.469 21949532 PMC3173668

